# Ab initio study of adsorption and diffusion of lithium on transition metal dichalcogenide monolayers

**DOI:** 10.3762/bjnano.8.270

**Published:** 2017-12-15

**Authors:** Xiaoli Sun, Zhiguo Wang

**Affiliations:** 1 School of Physical Electronics, University of Electronic Science and Technology of China, Chengdu, 610054, P.R. China

**Keywords:** anode materials, lithium adsorption, lithium diffusion, lithium ion batteries, transition metal dichalcogenide

## Abstract

Using first principles calculations, we studied the stability and electronic properties of transition metal dichalcogenide monolayers of the type MX_2_ (M = Ti, Zr, Hf, V, Nb, Ta, Mo, Cr, W; X= S, Se, Te). The adsorption and diffusion of lithium on the stable MX_2_ phase was also investigated for potential application as an anode for lithium ion batteries. Some of these compounds were found to be stable in the 2H phase and some are in the 1T or 1T' phase, but only a few of them were stable in both 2H/1T or 2H/1T' phases. The results show that lithium is energetically favourable for adsorption on MX_2_ monolayers, which can be semiconductors with a narrow bandgap and metallic materials. Lithium cannot be adsorbed onto 2H-WS_2_ and 2H-WSe_2_, which have large bandgaps of 1.66 and 1.96 eV, respectively. The diffusion energy barrier is in the range between 0.17 and 0.64 eV for lithium on MX_2_ monolayers, while for most of the materials it was found to be around 0.25 eV. Therefore, this work illustrated that most of the MX_2_ monolayers explored in this work can be used as promising anode materials for lithium ion batteries.

## Introduction

Lithium ion batteries (LIBs) have been widely used in portable electronic devices as power supplies, which have potential use in electrical vehicles (EVs) and smart grids. However, the energy and power density of current LIBs cannot satisfy the high demand of EVs. The development of new electrode materials is essential for improvement of the energy density. An ideal electrode material for LIBs should have good electronic conductivity, a lower Li diffusion energy barrier, as well as high energy and power densities. By reducing the bulk electrode materials to low-dimensional materials, a higher energy capacity and higher charge/discharge rate can be obtained as the low-dimensional materials have higher exposure to the electrolyte [1]. Two-dimensional materials, such as Co_3_O_4_, NiO, phosphorene, SnS and V_2_O_5_ all exhibit an excellent capacity retention, rate performance, lower energy barrier and long cycling life compared to their bulk counterparts used as electrode materials for LIBs [2–8].

Two-dimensional transition metal dichalcogenides, MX_2_ (where M and X correspond to transition metal and chalcogen atoms, respectively), have been synthesized using different strategies, such as exfoliation [9–10], physical vapour deposition [11] and chemical vapour deposition [12–14]. MX_2_ has received tremendous attention as an alternative to graphite for the anode material in LIBs [15–16]. In particular, MoS_2_ has been well-investigated as an anode material for LIBs both theoretically and experimentally. A graphene like-MoS_2_/graphene composite was shown to exhibit a high specific capacity of 1400 mA h/g and good rate performance as well as cycling ability [17]. It was reported that MoS_2_ zigzag nanoribbons are promising electrode materials for LIBs with a high power density and fast charge/discharge rates [18]. The presence of structural defects can enhance the adsorption of Li atoms onto two-dimensional materials. Different from the situation where Li atoms are trapped by the defects in graphene, the presence of structural defects does not affect the diffusion of lithium [19]. The main drawback of MoS_2_ is its poor electrical conductivity. Various strategies have been developed to improve the electrochemical properties of MoS_2_ as an anode for LIBs. Three-dimensional hierarchical structures constructed by assembling two-dimensional MoS_2_ nanosheets can deliver a capacity of 1009 mAh/g at 500 mA/g after 500 cycles [20]. The formation of composites of MoS_2_ with other materials, such as carbon-based materials and non-carbonaceous materials, can enhance the electromechanical properties of MoS_2_. Wang et al. [21] utilized a beneficial "bridging effect" of sulfur atoms to bind few-layered MoS_2_ with graphene, which provided fast electron conductivity and excellent cycling stability and superior rate performance. The composites exhibited a high discharge capacity of 1546 mAh/g after 300 cycles. The MoS_2_ composites grown on TiO_2_ nanotubes show better rate capability with a reversible capacity of 461 mAh/g at 1000 mA/g, compared with the capacity of pure MoS_2_ (129 mAh/g) at the same current density [22].

MX_2_ monolayers have three types of crystalline structures, hexagonal structure (2H), octahedral structure (1T) and distorted octahedral structure (1T') [23–25]. The structures depend on the arrangements of the M and X atoms. Phase transformation between the different phases occurs during the synthesis process and lithium/sodium intercalation [26–28]. Sun et al. [29] have studied the effect of electron doping on the stability of 2H- and 1T'-MoS_2_, and showed that electron doping can stabilize the crystal structure of 1T'-MoS_2_. The crystalline structure can also affect the energy conversion efficiency, for example in the hydrogen evolution reaction (HER). The basal plane of 2H-MoS_2_ is inert [30], where that of 1T'-MoS_2_ is catalytically active for HER [31]. Until now, there is no systematic study on the family of transition metal dichalcogenide monolayers used as anode for LIBs.

In this work, we studied the stability of MX_2_ monolayers, and the adsorption and diffusion of Li on the stable MX_2_ monolayers (M = Ti, Zr, Hf, V, Nb, Ta, Mo, Cr, W; X = S, Se, Te). These results are helpful for the design of two-dimensional transition metal dichalcogenide based electrodes for LIBs.

## Results and Discussion

**Figure 1:**
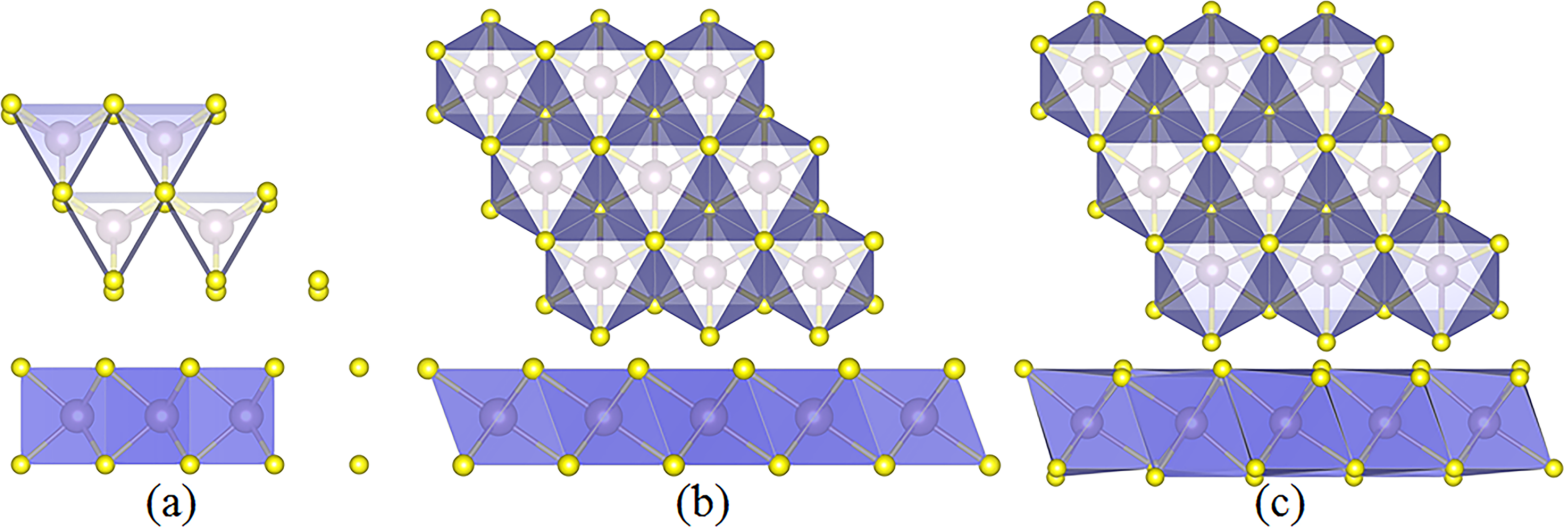
Top and side views of ball and stick models of a MX_2_ monolayer in (a) 2H, (b) 1T and (c) 1T' phase. The M atoms have octahedral and trigonal prismatic coordination in the 1T/1T' and 2H phase, respectively.

We systematically investigated the phase stability, Li adsorption and diffusion on MX_2_ monolayers (M = Ti, Zr, Hf, V, Nb, Ta, Cr, Mo, W; X = S, Se, Te). The combination of these elements have twenty seven possible binary compound materials. Three phases, including 2H, 1T and 1T' structures, were all considered for each of the binary monolayers. All the three structures can be viewed as a positively charged, two-dimensional M atoms, lattice-sandwiched by two hexagonal lattices of negatively charged X atoms. Each M atom is surrounded by six nearest X atoms, and each X atom is connected to three nearest M atoms with ionic M–X bonds. The side and cross-views of the ball and stick models of the MX_2_ monolayer are shown in Figure 1. The M atoms are located at the lattice positions of a hexagonal close-packed structure with a trigonal symmetry in the 2H-MX_2_ phase (Figure 1a), whereas M atoms are located at the octahedral/disordered octahedral centre of six S atoms in the 1T/1T' phase (Figure 1b,c). Some compounds are not stable in the 1T' phase, which will be relaxed to the 1T phase after relaxation.

The energy related to the 2H phase per formula unit (f.u.), *E* = *E*_1T_*_/_*_1T'_−*E*_2H_, is listed in Table 1. A negative value indicates that the 1T/1T' phase is more stable than the 2H phase. It can be seen from the table that some of these compound can be stable in the 2H phase, and some in the 1T or 1T' phase. Only a few of them are stable both in 2H/1T or 2H/1T' phases. The 2H phase is the minimum energy configuration for monolayers of NbX_2_ and TaX_2_, which agrees with previously reported results [32–33]. The energy of the 1T phase is smaller than the 2H phase for TiX_2_, VX_2_, VSe_2_, CrX_2_, ZrX_2_ and HfX_2_. 1T-VS_2_ monolayers are 0.02 eV/f.u. larger than the 2H phase, which indicates that the 1T phase is the energetically favourable one for these compounds, and VS_2_ monolayers maybe be stable both in the 2H/1T phase. The results agree with other calculations that imply that the 1T phase is more stable than 2H in TiX_2_ [32–34], CrS_2_ [35], ZrX_2_ [32–33] and HfX_2_ [33]. The 2H phase is the stable structure for MoS_2_, MoSe_2_, WS_2_ and WSe_2_ monolayers, which has also been predicted by other simulations [36–40]. 1T'-VTe_2_, 1T'-MoTe_2_ and 1T'-WTe_2_ are the energetically favourable phases. It is also can be seen from Table 1 that 1T'-MoTe_2_ is 0.06 eV/f.u. more energetically favourable than the 1T phase, which agrees with other simulations [36,39–40]. The energy difference between 2H-WTe_2_ and 1T'-WTe_2_ is 0.07 eV/f.u., which indicates MoTe_2_ and WTe_2_ maybe exist in two phases.

**Table 1:** Energy (eV) referenced to the 2H phase per formula unit. A negative value indicates that the 1T/1T' phase is more stable than the 2H phase.

M	S			Se			Te		
	2H	1T	1T'	2H	1T	1T'	2H	1T	1T'

Ti	0.00	−0.44	–	0.00	−0.33	–	0.00	−0.26	–
V	0.00	0.02	–	0.00	−0.16	–	0.00	−0.06	−0.15
Cr	0.00	−0.45	–	0.00	−0.39	–	0.00	−0.17	–
Zr	0.00	−0.54	–	0.00	−0.40	–	0.00	−0.26	–
Nb	0.00	0.21	–	0.00	0.22	–	0.00	0.15	0.10
Mo	0.00	0.68	0.60	0.00	0.35	0.29	0.00	−0.20	−0.26
Hf	0.00	−0.62	–	0.00	−0.50	–	0.00	−0.35	–
Ta	0.00	0.18	–	0.00	0.23	0.21	0.00	0.15	0.54
W	0.00	0.91	0.61	0.00	0.81	0.35	0.00	0.61	−0.07

The calculated lattice constants and bond length of the M–X bond in the stable phase is listed in Table 2 along with available values from other simulations. The values obtained in the present work agree well with other simulation results. It can be seen from Table 2 that lattice constants and bond lengths increase for the all the MX_2_ monolayers as the element X changes from S to Te in group VI for a given element M. The variation can be explained by the increasing atomic radius of elements X from S to Te.

**Table 2:** Lattice constants (*a*,*b*) and the bond length of the M–X bond (*d*_M-X_) in the stable phase as calculated in this work as compared to other values found in the literature from other simulations. The electronic conducting behaviour (ECB) of these compounds is also shown.

MX_2_	*a*/*b* (Å)	*d*_M-X_ (Å)	*a* (Å) [Ref.]	*d*_M-X_ (Å) [Ref.]	ECB

1T-TiS_2_	3.40	2.42	3.39 [33]	2.39 [41]	0.59
1T-TiSe_2_	3.56	2.57	3.53 [33]	2.51 [41]	0.29
1T-TiTe_2_	3.72	2.77	3.74 [33]	2.73 [41]	metal
2H-VS_2_	3.19	2.39	3.17 [42–44]	2.36 [42–44]	0.58
1T-VS_2_	3.25	2.38	3.18 [43,45]	2.35 [43–44,46]	metal
1T-VSe_2_	3.37	2.55	3.24 [41]	2.49 [46]	metal
1T'-VTe_2_	3.80/ 7.60	2.71/ 2.76/ 2.79/ 2.81	–	–	metal
1T-CrS_2_	3.33	2.41	–	–	metal
1T-CrSe_2_	3.47	2.56	–	–	metal
1T-CrTe_2_	3.67	2.81	–	–	metal
1T-ZrS_2_	3.57	2.55	3.68 [33,47]		0.92
1T-ZrSe_2_	3.70	2.68	3.79 [47]		0.29
1T-ZrTe_2_	3.89	2.90	3.98 [33]		metal
2H-NbS_2_	3.35	2.50	3.36 [48]	2.49 [48]	1.22
2H-NbSe_2_	3.49	2.64	3.48 [48]	2.62 [48]	1.00
2H-NbTe_2_	3.71	2.83	3.70 [48]	2.82 [48]	0.78
2H-MoS_2_	3.17	2.42	3.18 [47,49]	2.42 [48]	1.71
2H-MoSe_2_	3.32	2.55	3.32 [47]	2.55 [48]	1.41
1T-MoTe_2_	3.84	2.81	–	–	metal
1T'-MoTe_2_	3.89/ 7.88	2.53/ 2.55/ 2.60/ 2.61	–	–	0.12
1T-HfS_2_	3.57	2.54	3.64 [33]	–	1.09
1T-HfSe_2_	3.69	2.67	3.76 [33]	–	0.50
1T-HfTe_2_	3.88	2.87	3.97 [33]	–	metal
2H-TaS_2_	3.35	2.50	3.34 [48]	2.48 [48]	0.20
2H-TaSe_2_	3.47	2.63	3.48 [48]	2.62 [48]	0.46
2H-TaTe_2_	3.69	2.82	3.76 [48]	2.82 [48]	0.37
2H-WS_2_	3.18	2.44	3.18 [49]	2.42 [48]	1.96
2H-WSe_2_	3.32	2.57	3.32 [47–48]	2.55 [48]	1.66
2H-WTe_2_	3.56	2.76	–	–	1.22
1T'-WTe_2_	3.49/ 6.98	2.74/ 2.75/ 2.78/ 2.81	–	–	0.38

**Figure 2:**
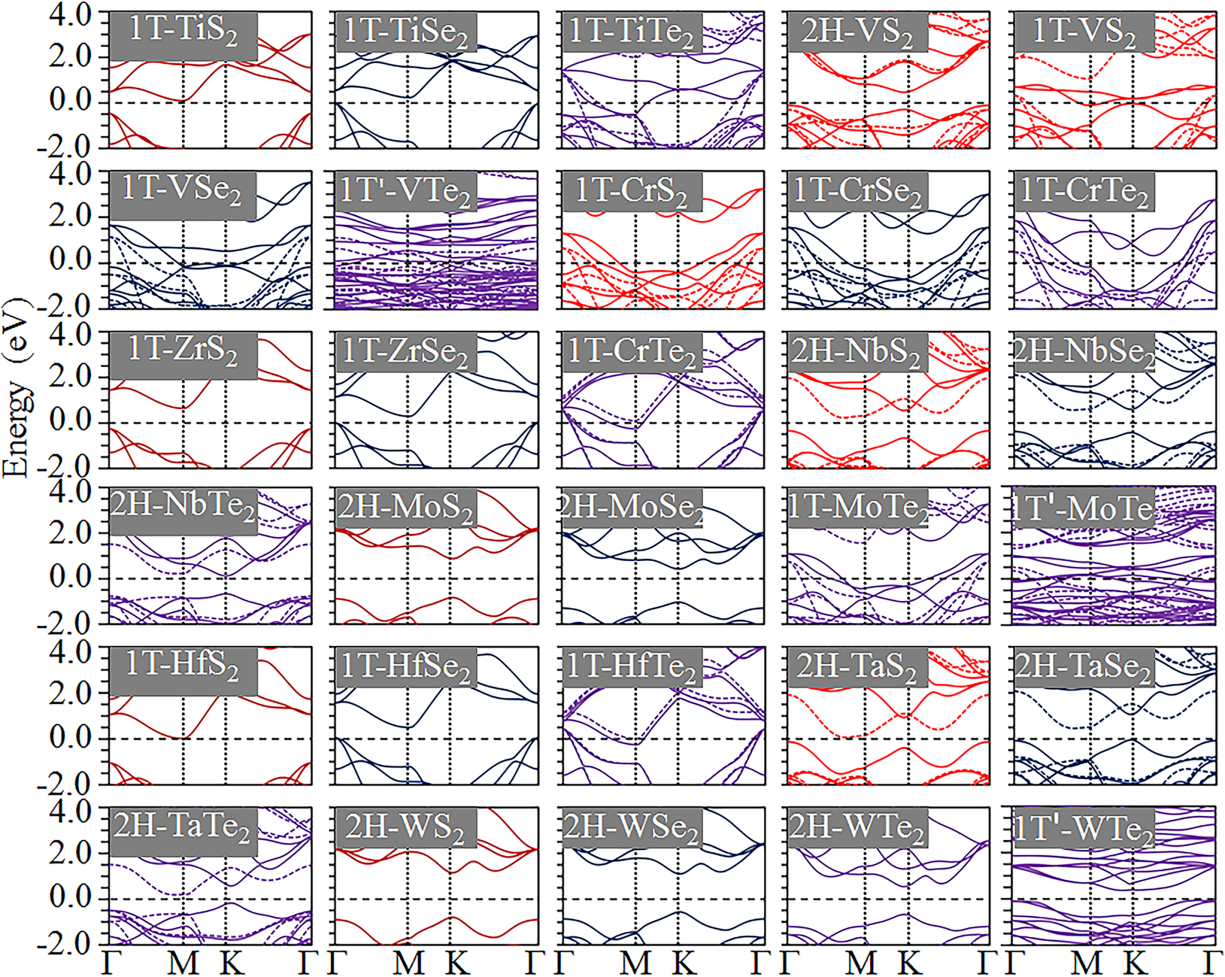
Band structures of MX_2_ monolayers in the stable phase. Fermi energy level is set to be 0.

The band structures of MX_2_ monolayers in the stable phase are shown in Figure 2. The MX_2_ monolayers can be semiconducting with a direct and indirect bandgap or metallic materials. The electronic conductive behaviour of these compounds are shown in Table 2. The 2H phase shows a semiconducting behaviour, such as 2H-WX_2_, 2H-NbX_2_, 2H-TaX_2_ and 2H-MoX_2_. The 1T phase can be metallic or semiconducting, such as in 1T-VX_2_ (X = S, Se), and 1T-CrX_2_ shows metallic behaviour, while 1T-TiX_2_, 1T-ZrX_2_ and 1T-HfX_2_ show semiconducting and metallic behaviour with X = S/Se and X = Te, respectively. 1T'-VTe_2_ and 1T'-MoTe_2_ show metallic behaviour and 1T'-WTe_2_ has a narrow bandgap of 0.50 eV. The obtained bandgap values are close to those previously reported for TiS_2_ [32], CrTe_2_ [40], TiX_2_ [32–33,47], MoX_2_ [33,40,47,50–55], HfX_2_ [33,47] and WX_2_ [33,40,47,50,55–58]. The metallic MX_2_ monolayers have good electrical conductivity, which may make them good anode materials.

**Figure 3:**
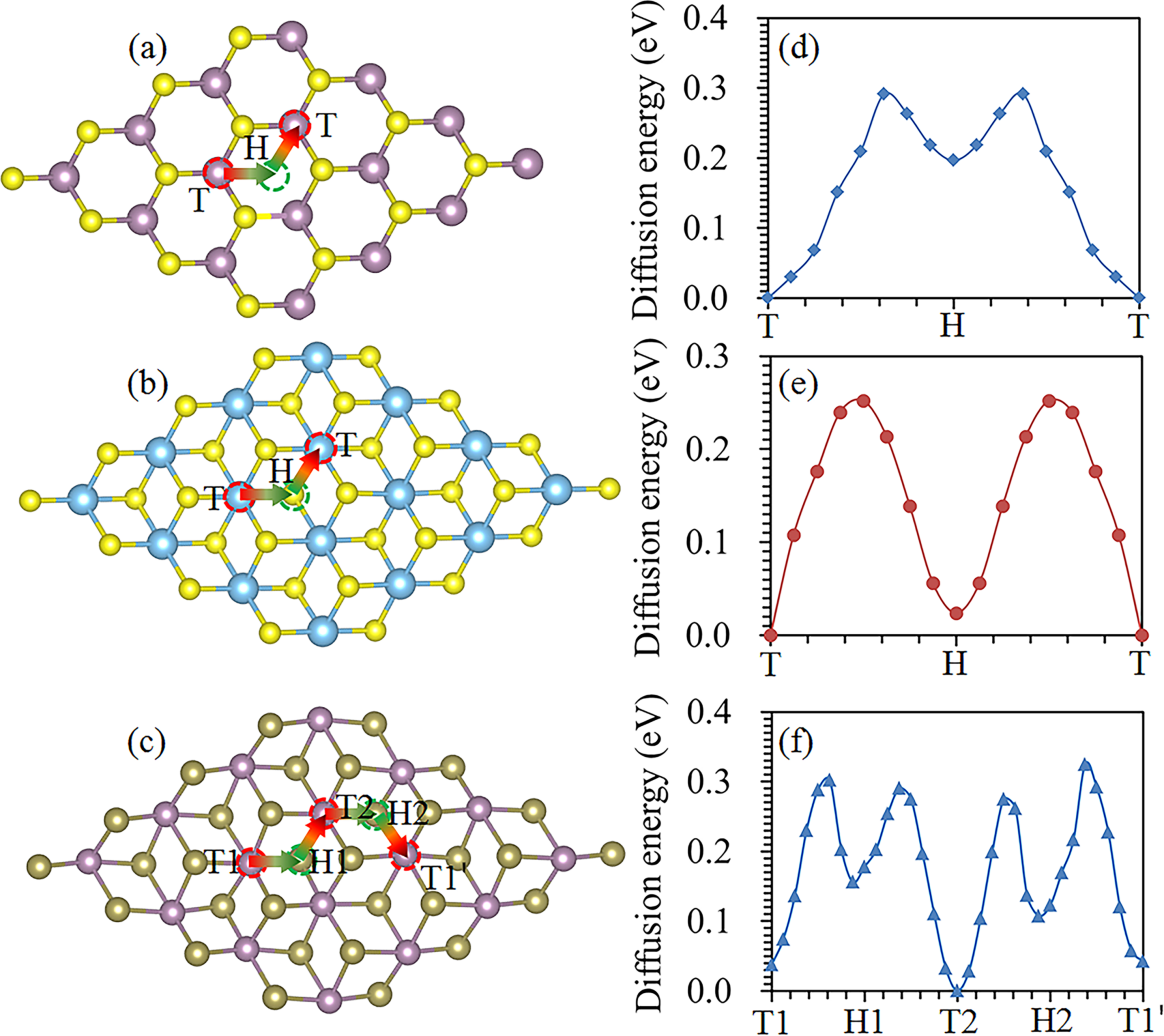
Possible adsorption sites and diffusion paths for Li on a monolayer of (a) 2H-, (b) 1T- and (c) 1T'-MX_2_. Diffusion energy profiles for Li on (d) 2H-MoS_2_, (e) 1T-TiS_2_, and (f) 1T'-MoTe_2_.

**Figure 4:**
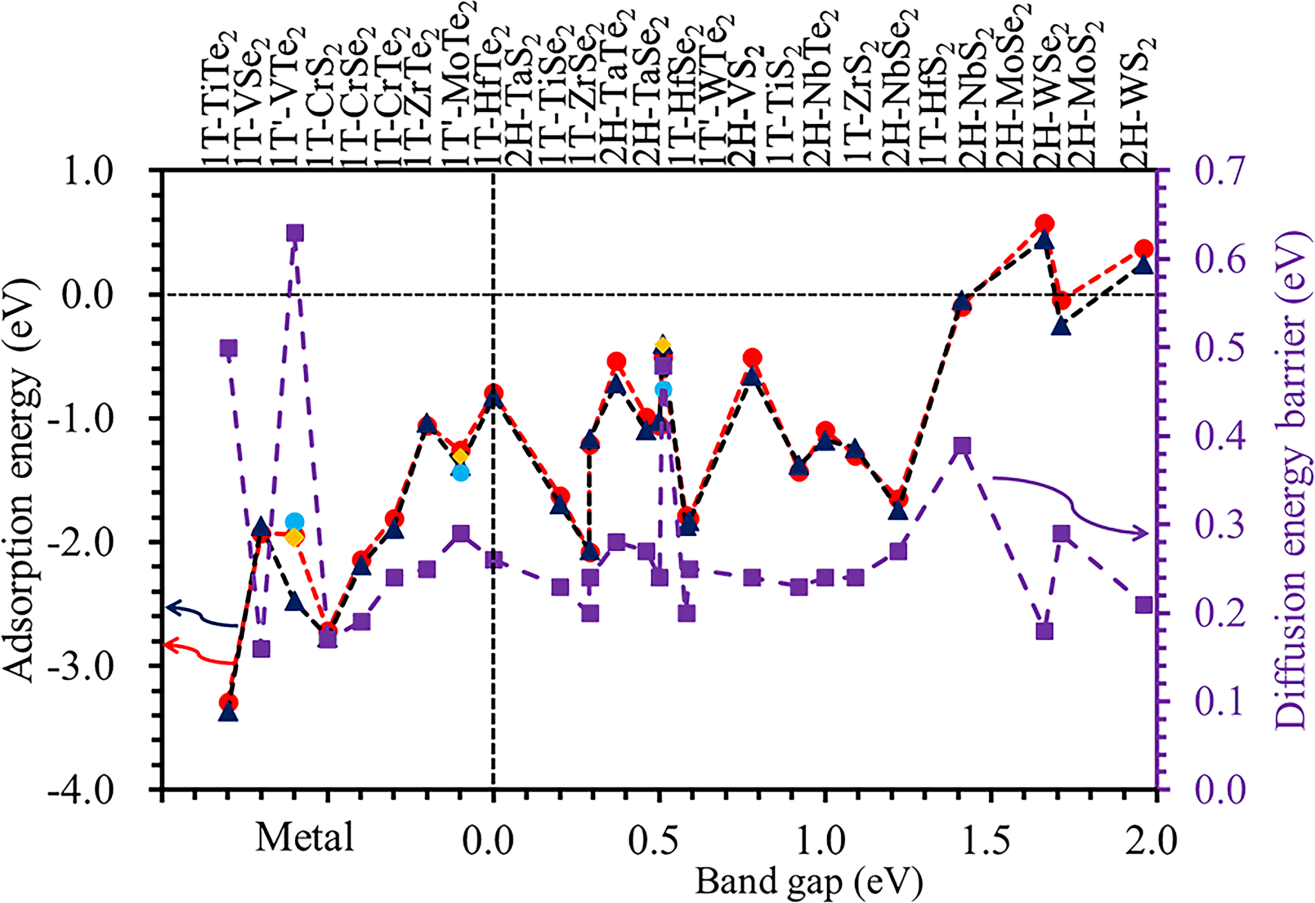
Adsorption energy and diffusion energy barrier for Li on MX_2_ monolayers in the stable phase.

As shown in Figure 3a and Figure 3b, there are two stable adsorption sites, that is, the hollow site (H) and the top position above the M atom (T) for Li to be adsorbed on the 2H- and 1T-MX_2_ monolayers [18]. Four adsorption sites (T1, T2, H1, and H2) are considered for Li adsorption onto the 1T'-MX_2_ monolayer, as shown in Figure 3c. To analyse the stability of Li adsorbed on the MX_2_ monolayers, the adsorption energy, *E*_ad_(Li), is calculated using Equation 1:

[1]
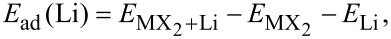


where *E*_MX2+Li_ and *E*_MX2_ are the total energy of the MX_2_ monolayer with and without Li adsorption, respectively. *E*_Li_ is the energy of a Li atom in bulk material. The calculated adsorption energy of Li on the stable phase of the MX_2_ monolayers is shown in Figure 4. The adsorption energy has positive values for Li adsorbed on 2H-WS_2_ and 2H-WSe_2_, which indicates that Li cannot be adsorbed on these two compounds and they are not ideal anodes for LIBs. The other compounds have negative values of adsorption energy. The adsorption energy of Li on 2H-MoS_2_ is −0.05 and −0.25 eV for H and T sites, respectively. The materials will have a large energy storage capacity if they have a large exothermic reaction energy with Li [19]. Previous studies have shown that the 2H-MoS_2_ monolayer is a good anode material for LIBs [26–28]. The absolute value of the adsorption energy for Li adsorbed on other compounds is larger than that of 2H-MoS_2_, so other MX_2_ compounds are also good anode candidates for LIBs. The adsorption energy as a function of the bandgap of the MX_2_ monolayer is show in Figure 4. It can also be seen from the figure that MX_2_ is a semiconducting material with a narrow bandgap, and for metallic materials, the adsorption energy has larger negative values. The materials with a large bandgap have smaller adsorption energy, even those with positive values. For example, the bandgap energies are 1.96, 1.71, 0.78, 0.58 and 0.29 eV and the adsorption energies for Li adsorbed at H/T sites are 0.37/0.24, −0.05/−0.25, −0.51/−0.65, −1.79/1.87 and −2.08/2.07 eV on 2H-WS_2_, 2H-MoS_2_, 2H-NbTe_2_, 2H-VS_2_ and 1T-TiSe_2_ monolayers, respectively.

The diffusion of Li on the MX_2_ monolayers is through the T→H→T and T1→H1→T2→H2→T1 paths for the 2H/1T and 1T' phases, respectively [59], as shown in Figure 3a–c. The typical diffusion energy profiles are shown in Figure 3d, Figure 3e and Figure 3f for Li on 2H-MoS_2_, 1T-TiS_2_, and 1T'-MoTe_2_ monolayers, respectively. The constrained method was used to evaluate the diffusion behaviour of Li on MX_2_ monolayers – this method is more simple and intuitive compared to the nudged elastic band method and dimer method [60]. The diffusion energy barriers are 0.29, 0.25 and 0.28 eV for Li on 2H-MoS_2_, 1T-TiS_2_, and 1T'-MoTe_2_ monolayers, respectively. These values are reasonable for use as anodes for LIBs. The Li diffusion energy barrier on a MX_2_ monolayer is shown in Figure 4. Our calculated values agree well with those reported by other researchers. The diffusion energy barrier for Li on the 2H phase of monolayer WS_2_, WSe_2_ and VS_2_ is 0.21 eV, 0.18 eV and 0.20 eV, respectively, which is consistent with the previously reported values of 0.22, 0.23 eV [61], and 0.22 eV [42] respectively. The diffusion energy barrier of Li on 2H-MoS_2_ monolayer is 0.29 eV, which is consistent with the previously reported value of 0.25 eV [18,42].

A good anode material should have a high electron and Li mobility and a large exothermic reaction energy with lithium. High electronic and ion mobility determine the rate capability and cycling performance, and a large exothermic reaction energy indicates the anode materials have a large energy storage capacity. The diffusion energy barrier is in the range between 0.17 and 0.63 eV, and most are around 0.25 eV, which indicates that all the MX_2_ monolayers have a reasonable diffusion energy barrier for lithium. The metallic MX_2_ monolayers and those with small bandgaps have a large adsorption energy for Li, which indicates that they are good anode materials for LIBs with high electronic and ion mobility and large energy storage capacity.

## Conclusion

Using density functional theory (DFT) simulations, the stability and electronic properties of MX_2_ monolayers were investigated. TiX_2_, VSe_2_, CrX_2_, ZrX_2_ and HfX_2_ are energetically favourable in the 1T phase, and 1T-VS_2_ can be stable both in the 2H/1T phase. The 2H phase is the stable structure for MoS_2_, MoSe_2_, WS_2_ and WSe_2_. The 1T' phase is the most energetically favourable for VTe_2_, MoTe_2_ and WTe_2_. The 2H phase shows a semiconducting behaviour, for example, 2H-WX_2_, 2H-NbX_2_, 2H-TaX_2_ and 2H-MoX_2_. The 1T phase can be metallic or semiconducting, for example 1T-CrX_2_ shows a metallic behaviour while 1T-TiX_2_, 1T-ZrX_2_ and 1T-HfX_2_ show semiconducting and metallic behaviour with X = S/Se and X = Te, respectively. 1T'-VTe_2_ and 1T'-MoTe_2_ show metallic behaviour and 1T'-WTe_2_ has a narrow bandgap of 0.50 eV. The adsorption and diffusion of lithium on the stable MX_2_ phase were also investigated. The results show that lithium is energetically able to adsorb on MX_2_ monolayers, which are semiconductors with a narrow bandgap, and on metallic materials. Lithium cannot be adsorbed on 2H-WS_2_ and 2H-WSe_2_, which have a large bandgap of 1.66 and 1.96 eV, respectively. The diffusion energy barrier is in the range between 0.17 and 0.63 eV for lithium on MX_2_ monolayers, and most of the materials are around 0.25 eV. It is therefore concluded that most of the MX_2_ monolayers can be used as promising anode materials for lithium ion batteries.

## Simulation Details

All the spin-polarized DFT calculations were performed with SIESTA code [62], in which norm-conserving pseudopotentials and a Perdew–Burcke–Ernzerhof functional was used to describe the electron–ion interaction and electron exchange correlation, respectively. Numerical atomic orbits were represented as double zeta basis sets plus polarization, and a cut-off energy of 250 Ry was chosen to calculate the Hamiltonian element. The Monkhorst–Pack scheme with 11 × 11 × 1 *k*-point meshes were used for integration in the irreducible Brillouin zone for the relaxation of the primitive cell. A 2 × 2 × 1 *k*-point mesh was used for the calculation of adsorption and diffusion of Li on a 6 × 6 × 1 supercell. As the electrochemical process involves insertion of Li ions into anode materials with a concurrent flow of electrons to compensate charge balance, and therefore, the neutral state of Li was considered in this work. The atomic positions were relaxed by using a conjugate gradient minimization until the Hellmann–Feynman force is less than 0.02 eV/Å on each atom. A vacuum spacing between the slabs and its image of greater than 20 Å is given to avoid the periodic image interactions. As the radii are different for different elements of X in MX_2_ monolayers (i.e., the radius increases from S to Te in group VI), the lattice constants and bond length of the M–X bond will change for MX_2_ monolayers with different X elements, which can affect the adsorption and diffusion of Li on MX_2_ monolayers.
